# The interaction of botanicals and AMPK signaling in the inhibition of castrate-resistant prostate cancer

**DOI:** 10.1186/2049-3002-2-S1-P85

**Published:** 2014-05-28

**Authors:** Jay Whelan, Yi Zhao

**Affiliations:** 1 Nutrition, University of Tennessee, Knoxville, Tennessee, USA

## Background

Reprogramming of metabolic pathways and abnormal acetylation of DNA are two common features of cancer, including prostate cancer (PCa).

## Materials and methods

CWR22Rv1 cells (a castrate-resistant human PCa cell line) were treated with a polyherbal mixture of botanicals (PHBs) consisting of 10 herbal extracts [1] and we investigated whether inhibition of cell proliferation was linked to the activity of 5’adenosine monophosphate (AMP)-activated protein kinase (AMPK), a potential tumor suppressor protein that functions as a central energy sensor within the cell. In addition, we investigated whether this inhibition was also the result of synergy among the bioactives as determined by the Chou-Talalay method [2].

## Results

Our results show that the PHBs can modify lipogenesis in CWR22Rv1 cells by enhancing the activation of AMPK, with concomitant decreases of *de novo* lipid biosynthesis through the phosphorylation of acetyl-CoA carboxylase and by inhibiting the expression of fatty acid synthase and SREBP1c. Also via AMPK, the PHBs inhibit the activity of mTORC1 complex by phosphorylating raptor, decreasing the activity of S6K. These effects can be mimicked by treatment of AICAR (activator of AMPK) and over-expression of AMPKalpha, and attenuated by compound C (AMPK inhibitor) and siRNA silencing of AMPK. Furthermore, the PHBs increase the cellular pools of acetyl CoA via mechanisms that may include ATP-citrate lyase. Coordinately, there are increases in acetylation of histone 3 and expression of the tumor suppressor proteins p21 and p27, a mechanism that most likely involves the down regulation of class I and II histone deacetylases and up regulation of histone acetyl transferase (i.e., CBP/p300) via ERK1/2 signaling. The inhibitory effects on cell proliferation can be attributed in part to the synergistic activity of some of the individual components in the PHBs as determined by the Chou-Talalay method [2] lowering the effective dose/concentrations from micromolar to more physiologically-relevant nanomolar levels *in vitro*.

## Conclusions

Bioactive nutrients derived from multiple herbs inhibit the growth of androgen-deprived castrate resistant PCa cells via multiple mechanisms, one of which involves up regulation of AMPK signaling.
